# Polyamines in Food

**DOI:** 10.3389/fnut.2019.00108

**Published:** 2019-07-11

**Authors:** Nelly C. Muñoz-Esparza, M. Luz Latorre-Moratalla, Oriol Comas-Basté, Natalia Toro-Funes, M. Teresa Veciana-Nogués, M. Carmen Vidal-Carou

**Affiliations:** ^1^Department of Nutrition, Food Sciences and Gastronomy, Faculty of Pharmacy and Food Sciences, University of Barcelona (UB), Barcelona, Spain; ^2^Research Institute of Nutrition and Food Safety of the University of Barcelona (INSA·UB), Barcelona, Spain; ^3^Catalonian Reference Network on Food Technology (XaRTA), Barcelona, Spain; ^4^Eurecat, Technological Unit of Nutrition and Health, Technology Centre of Catalonia, Reus, Spain

**Keywords:** spermidine, spermine, putrescine, polyamines, human health, food, breast milk

## Abstract

The polyamines spermine, spermidine, and putrescine are involved in various biological processes, notably in cell proliferation and differentiation, and also have antioxidant properties. Dietary polyamines have important implications in human health, mainly in the intestinal maturation and in the differentiation and development of immune system. The antioxidant and anti-inflammatory effect of polyamine can also play an important role in the prevention of chronic diseases such as cardiovascular diseases. In addition to endogenous synthesis, food is an important source of polyamines. Although there are no recommendations for polyamine daily intake, it is known that in stages of rapid cell growth (i.e., in the neonatal period), polyamine requirements are high. Additionally, *de novo* synthesis of polyamines tends to decrease with age, which is why their dietary sources acquire a greater importance in an aging population. Polyamine daily intake differs among to the available estimations, probably due to different dietary patterns and methodologies of data collection. Polyamines can be found in all types of foods in a wide range of concentrations. Spermidine and spermine are naturally present in food whereas putrescine could also have a microbial origin. The main polyamine in plant-based products is spermidine, whereas spermine content is generally higher in animal-derived foods. This article reviews the main implications of polyamines for human health, as well as their content in food and breast milk and infant formula. In addition, the estimated levels of polyamines intake in different populations are provided.

## Introduction

**Figure 1:**
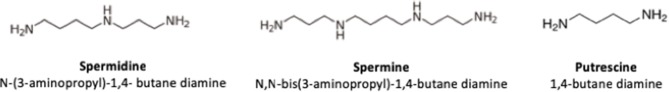
Chemical structure of polyamines.

In 1678, Antoni van Leeuwenhoeck discovered the presence of crystals in human semen, which 200 years later (1888) were named spermine by A. Landenburg and J. Abel. The chemical structure of spermine and spermidine was determined in 1924 (1). The polyamines spermidine (N-(3-aminopropyl)-1,4-butane diamine), spermine (N,N-bis (3-aminopropyl)-1,4-butane diamine), and putrescine (1,4-butane diamine) have a low molecular weight and are characterized by having two or more amino groups. They are found in all living cells, including in microorganisms, plants, and animals. Due to their structure (Figure 1), polyamines are relatively stable compounds, capable of resisting acidic and alkaline conditions and they can establish hydrogen bond with hydroxyl solvents such as water and alcohol (2–6). In the organism, at physiological pH they are completely protonated and strongly bound to polyanionic macromolecules such as DNA and RNA (2, 7, 8). On the other hand, polyamines can also be found in food of both animal and plant origin. An important source of polyamines for humans is breast milk and infant formula (2, 4).

### Polyamines and Health

Polyamines play an essential role in cell growth and proliferation, the stabilization of negative charges of DNA, RNA transcription, protein synthesis, the regulation of the immune response, apoptosis, the regulation of ion channels, particularly by blocking potassium channels, and as antioxidants (2, 4, 5, 7, 9–12).

The antioxidant activity of polyamines mainly affects membrane lipids and nucleic acids. Spermine is the polyamine with the strongest antioxidant properties, associated with its higher number of positive charges. The main mechanism of polyamine antioxidant action is metal chelation, which prevents the formation of hydroperoxides and delays the generation of secondary oxidation compounds (13–16). It has also been proposed that polyamines can eliminate free radicals, especially in lipophilic media (14, 16).

### Polyamine Homeostasis

**Figure 2:**
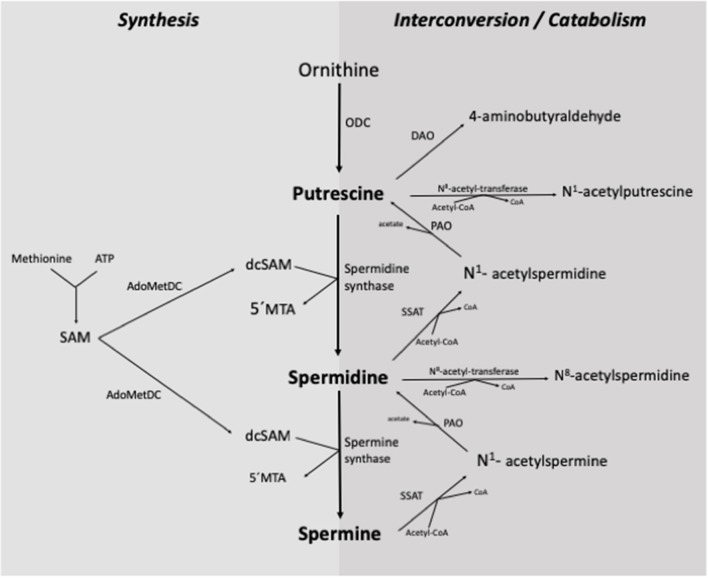
Synthesis and interconversion/catabolism of polyamines in the organism. ODC, ornithine-decarboxylase; SSAT, spermidine/spermine N^1^-acetyl-transferase; PAO, polyamine-oxidase; DAO, diamine-oxidase; SAM, S-adenosyl-methionine; AdoMetDC, S-adenosyl-L-methionine-decarboxylase; dcSAM, decarboxylated S-adenosyl-methionine; 5'MTA, 5'-methylthioadenosine; Acetyl-CoA, Acetyl coenzyme-A [Adapted from (7) and (2)].

The *de novo* synthesis of polyamines in the organism begins with the formation of putrescine from the amino acid ornithine, catalyzed by the enzyme ornithine decarboxylase (ODC) (Figure 2). Putrescine is converted to spermidine by spermidine synthase through the addition of a propylamine group derived from the decarboxylation of S-adenosyl-methionine. Subsequently, spermidine is transformed into spermine by spermine synthase, which adds a second propylamine group (2, 4, 7, 12, 17).

The interconversion of polyamines is a cyclic process that controls their turnover and regulates intracellular homeostasis (Figure 2). This process begins with the acetylation of any of the three polyamines, which is catalyzed by an N-acetyl-transferase enzyme with the participation of acetyl coenzyme-A. Subsequently, the enzyme polyamine oxidase (PAO) removes a propylamine group, and putrescine is obtained from the acetylated metabolite of spermidine, or spermidine from the acetylated metabolite of spermine (2, 10, 12, 17, 18).

The elimination of polyamines from the organism is carried out by the oxidative deamination of a primary amino group, mainly by the action of diamine oxidase (DAO) and PAO. Both enzymes can act on polyamines and their acetylated derivatives (2, 4, 7, 10, 17).

Besides endogenous synthesis, polyamines also have an exogenous origin, mainly food and breast milk (2). In addition, gut microbiota is also described as a source of polyamines, mainly forming in the large intestine (2, 19, 20). Some recent studies have been linked different intestinal microbial species with the synthesis of these compounds (20). However, more information is still needed on the capability to form polyamines of the gut microbiota and the corresponding biosynthetic pathways. Finally, intestinal and pancreatic secretions and catabolism products of intestinal cells also contribute to the polyamines in the gut (2). Polyamines are absorbed in the duodenum and in the first portion of the jejunum by various mechanisms, including transcellular (through passive diffusion and transporters) and paracellular pathways (2, 4, 21). Polyamines are partly metabolized in the intestinal wall before reaching the blood circulation, and those that pass into the circulation are distributed throughout the organism and captured by the tissues, where they can undergo interconversion reactions.

The highest concentrations of polyamines are found in the intestine, thymus and liver (2, 4). A diet enriched with polyamines raises plasma levels in experimental animals and humans (22).

### Potential Effects of Polyamines

#### Postnatal Stage

Several studies describe the importance of polyamines in humans, especially in the early stages of life. It is known that during rapid cell growth, particularly in the neonatal stage, the need for polyamines increases (4, 5, 21, 23, 24). Requirements are also higher after surgery or during periods of wound-healing and aging (2, 23, 25).

Polyamines (spermine and spermidine) promote the proliferation and maturation of the gastrointestinal tract and are involved in the differentiation and development of the immune system (5, 21, 25–31). In addition, due to their antioxidant properties, these compounds can participate in the regulation of the inflammatory response (12, 22).

Several studies have demonstrated that oral administration of polyamines in mice induces early postnatal maturation of the intestine and acts in the repair of the intestinal mucosa and in the immune and inflammatory response. Spermine and spermidine modified protein expression and the activity of disaccharidases and accelerated postnatal intestinal maturation, producing morphological changes in the intestinal epithelium and mucosal permeability (29). They also participate in the maturation of associated organs such as the liver and pancreas. In another study with mice, the oral administration of polyamines, mainly spermidine, was found to promote the early maturation of glycoprotein fucosylation. A dose of 10 μmol/day of each polyamine increased the activity of α-1,2-fucosyltransferase and α-L-fucosidase and induced the synthesis of α-1,2-fucoprotein (32, 33). The authors of this study suggest that postnatal changes in the fucosylation of intestinal glycoproteins could be related mainly to the intake of polyamines, especially spermidine and spermine. Another study showed that oral administration of spermine in mice increases the activity of alkaline phosphatase and disaccharidase, and subsequently alters intestinal maturation (34). The administration of spermine and spermidine in newborn rats increased the intestinal weight and length and accelerated its maturation (21). Regarding the immune response at the intestinal level, various studies in animals have indicated that the oral administration of spermine and spermidine in the postnatal period improves the maturation of the intestinal immune cells and increases the levels of immunoglobulin A in the villi and crypts of the intestine (21, 29).

In humans it is widely reported that breast milk enhances the maturation of immune cells and decreases intestinal permeability to antigenic macromolecules, reducing the risk of food hypersensitivity in the infant (21, 25, 26, 29, 35, 36).

#### Aging

In the aging process, the cellular levels of spermine and spermidine and the enzymatic activity of ODC tend to decrease (17, 37, 38). Enrichment of the diet with polyamines during this stage can reduce the risk of age-associated pathologies and promote longevity (39, 40). In a study in aging mice, a diet with high levels of spermine and spermidine (374 and 1,540 nmol/g, respectively) increased the concentrations of these compounds in the blood and reduced levels of pro-inflammatory markers, age-associated DNA methylation, renal glomerular atrophy and mortality (39). It has also been observed that spermidine increases autophagy, which involves the removal of damaged proteins and organelles from cells, thus inhibiting the aging process (6, 41–43). In a follow-up study of a cohort of 829 participants during 20 years, spermidine showed the strongest inverse relation with mortality among 146 nutrients investigated. This effect was dose-dependent, and the authors explain that spermidine effectively induced autophagy and can reduce the acetylation of histones, which are critical processes for cell homeostasis in aging. In this sense, a diet rich in spermidine, mainly from foods of vegetable origin (green pepper, wheat grain, mushrooms, etc.), was associated with a decrease in the risk of all-cause mortality in the general community (42).

#### Cardiovascular Disease

The antioxidant and anti-inflammatory effects attributed to polyamines can play an important role in the prevention of chronic inflammatory pathologies, such as cardiovascular diseases (22). A higher intake of spermidine has been correlated with a lower incidence of cardiovascular diseases and a decrease in blood pressure and heart failure (44). It is likely that the anti-inflammatory role of polyamines in the prevention and treatment of cardiovascular disease is similar to that of polyunsaturated fatty acids (PUFA 3-n) and statins (22, 45). In animal studies, mainly in aging mice, spermidine has been shown to decrease age-induced arterial stiffness and oxidative damage of endothelial cells (44). In addition, 6 week supplementation of spermine and spermidine in mice reversed age-associated changes in myocardial morphology (myocardial fibrosis) and inhibited cellular apoptosis of the heart (46).

#### Diabetes

Glycation has an important role in the development of diabetes complications, so compounds that can counteract this reaction are desirable. Due to their chemical structure, polyamines could function as antiglycan agents, delaying the accumulation of advanced glycation end-products (AGEs) (7, 47). This effect would be due to the interaction between the free amino groups of polyamines and the highly reactive carbonyl compounds (10, 18). *In vitro* studies have demonstrated that the millimolar concentrations of spermine present in the cell nucleus can protect DNA and histones from glycation (18).

On the other hand, some authors have observed a higher PAO activity in children with diabetes mellitus type 1, which could induce an increased production of free radicals and subsequent oxidative damage (47). Therefore, more studies are needed to clarify the role of polyamines in diabetes and establish recommended levels of polyamine intake for the diabetic population.

#### Cancer

Elevated levels of polyamines in cancer patients are associated with tumor growth (48, 49). A deregulation in polyamines biosynthesis, mainly due to an increase in the activity of the ODC enzyme, leads high intracellular polyamine content in cancer cells (12, 39, 45, 49). Therefore, controlling polyamine synthesis could be useful in antineoplastic therapy. According to different experimental studies and clinical trials, the combined treatment using difluoro-methylornithine (DFMO), a potent and irreversible inhibitor of ODC, with polyamine transport inhibitor drugs or non-steroidal anti-inflammatory drugs (NSAIDs), efficiently reduced the carcinogenesis by inhibiting polyamines synthesis and stimulating polyamines catabolism and export (48, 49).

An increase of the acetylated metabolites of polyamines has been observed in urine or blood in patients suffering cancer disease. The rise of acetylated polyamines in urine may be explained by an increase of cellular polyamines, an increase of the SSAT activity, a major excretion of acetylated metabolites from cells or by a decrease of their oxidative degradation by PAO enzyme, although the molecular mechanisms are not well-elucidated (50). The development of more sensitive metabolomic techniques in the last decade has allowed detailed polyamine metabolic profiles to be associated with certain types of cancer (48). In fact, increased levels of acetylated polyamines in urine or blood, particularly, N1,N12-diacetylspermine, N1,N8-acetylspermidine, N1-acetylspermine, and N8-acetylspermidine have been found in patients with ovarian, prostate, colorectal, pancreatic, breast and lung cancers. Among them, N1,N12-diacetylspermine has been extensively described as the most effective urinary biomarker for several types of cancer and to monitor tumor's progression (48, 51, 52).

Despite advances in understanding the role of polyamines in cancer, more research is required on the molecular basis in which polyamines participate. Determining how to optimally intervene in polyamine metabolism and function could lead to therapeutic benefits in cancer treatment.

### Polyamines in Food

Polyamines are found in foods of both animal and plant origin, either in a free or conjugated form. Conjugated polyamines are found in plant-derived foods mainly linked to phenolic compounds (4, 24). In foods, spermidine and spermine are primarily naturally present, coming from raw plant and animal tissues, whereas putrescine may also be formed by the activity of fermentative or contaminating microorganisms (12, 53). It has also been described that spermidine and spermine may partly have a bacterial origin, especially in fermented products (12, 54, 55). Therefore, processing and storage conditions can influence the total content of polyamines.

#### Breast Milk and Infant Formula

The first dietary exposure to polyamines is through breast milk. Table 1 shows the contents of polyamines in breast milk and infant formula reported in the literature, with all results expressed in nmol/ml to facilitate comparison. All the studies reviewed agree that the content and profile of these compounds can vary depending on factors such as genetics, the lactation phase, and the age, nutritional status and dietary intake of the mother.

**Table 1:** Average contents of polyamines in breast milk and infant formula.

	**Putrescine nmol/ml**	**Spermidine nmol/ml**	**Spermine nmol/ml**	**References**
Full-term breast milk	0.896	3.849	3.440	(26)^a^
	0.615	3.512	4.490	(56)^b^
	0.238	2.196	3.128	(27)
	0.030	0.124	0.104	(31)^b^
	0.851^1^	4.138^1^	1.596^1^	(11)^a^
	0.726^2^	3.470^2^	1.463^2^	
	0.824	4.578	1.735	(25)^c^
	0.658	3.993	4.077	(57)
	0.204	3.520	5.080	(5)^b^
*Range*	0.030–896	0.124–4.578	0.104–5.080	
Pre-term breast milk	0.058	0.462	0.302	(31)^b^
	1.655	6.151	1.677	(25)^c^
First formula	14.300	0.186	0.129	(26)
	0.018	0.187	nd	(56)
	0.374	0.912	0.675	(27)
	3.880	2.265	0.363	(25)
	3.596	0.516	0.302	(57)
	10.323	6.933	7.339	(58)
*Range*	0.018–14.300	0.186–6.933	0.129–7.339	
Follow-on formula	12.796	0.138	0.158	(26)
	0.263	1.198	0.458	(27)
	5.349	2.382	0.363	(25)
	7.533	4.241	6.227	(58)
*Range*	0.263–12.796	0.138–4.241	0.158–6.227	
Pre-term formula	1.057	0.215	0.172	(56)
	15.451	4.331	0.623	(25)

1 Milk from mothers of normal weight.

2 Milk from obese mothers.

a Breast milk 2 months postpartum.

b Breast milk 1 month postpartum

c Breast milk 1 week postpartum.

*nd, not detected*.

The major polyamines in breast milk are spermidine and spermine and their contents differ considerably, with coefficients of variation >68 and 53%, respectively. Spermine values are generally higher, except in two studies by the same author, in which higher values are reported for spermidine (11, 25). As indicated in Table 1, the breast milk analyzed in different studies corresponds to different phases of lactation, which could contribute to the high variability observed. In this sense, some authors have described that the polyamine content tends to decrease over the course of lactation (26, 56). Additionally, two studies found higher polyamine contents in the milk of mothers of preterm infants compared to full-term (25, 31). Also, as preliminary information it should be noted that milk from obese mothers was found to contain fewer polyamines than milk from those with normal weight (11).

In infant formula the variability among results of different studies is even higher than for breast milk, with coefficients of variation of 89% for putrescine, 116% for spermidine, and 160% for spermine. Despite this variability, it can be extrapolated that the polyamine content and profiles in infant formula differ from those of breast milk. For example, the major polyamine in infant formula is putrescine, its content usually higher than in breast milk, whereas spermidine and spermine levels tend to be lower. When first and follow-on formula are compared, no differences can be observed in the mean contents of polyamines. Likewise, the few available data on polyamines in infant formulas for premature babies do not allow to observe differences with other types of formulas.

The available data on polyamine content in breast milk and infant formula are scarce and, in some cases, outdated. More studies are needed to clarify whether the variability observed both in breast milk and infant formula is due to the use of different analytical methodologies or to other factors that have not been sufficiently investigated.

#### Food of Plant Origin

Polyamines are ubiquitous in foods of plant origin, although their content and distribution vary depending on the type of food (Table 2). Spermidine, present in all plant-derived foods, is generally the predominant polyamine. The food categories with the highest contents of spermidine and spermine are cereals, legumes and soy derivatives. Wheat germ and soybeans stand out in particular, with respective values of 2,437 and 1,425 nmol/g for spermidine and 722 nmol/g and 341 nmol/g for spermine (37, 59). Mushrooms, peas, hazelnuts, pistachios, spinach, broccoli, cauliflower and green beans also contain significant amounts of both polyamines. The lowest levels are found in the fruit category. For example, in apples, pears, cherries, oranges or tangerines, reported values for spermidine are lower than 21 nmol/g and <1.98 nmol/g for spermine.

**Table 2:** Ranges of average polyamine content (nmol/g) in foods of plant origin.

**Food categories**	**Putrescine**	**Spermidine**	**Spermine**	**References**
**Fruits**				
Apple, avocado, banana, cherry, kiwi, mandarin, orange, pear, peach, pineapple, strawberry, fruit juices.	nd−1,554	6.9–98	nd−25	(9, 37, 59, 60)
**Vegetables**				
Broccoli, cabbage, cauliflower, carrot, celeriac, courgette, cucumber, eggplant, green beans, green pepper, lettuce, mushroom, onion, potato, spinach, tomato.	5.7–794	6.9–398	nd−54	(9, 37, 59–69)
**Legumes and soybean products**				
Chickpeas, lentils, peas, white beans, red kidney beans, soybean, soybean sprouts, soybean milk, tofu, soy sauce, miso	nd−525	1.0–1,425	nd−341	(37, 55, 59, 61, 63, 64, 66–68, 70)
**Nuts and oilseeds**				
Almonds, chestnuts, pistachios, seeds	34–488	41–383	63–165	(37, 64)
**Cereals**				
Rice, wheat germ, white bread	2.3–704	2.8–2437	nd−722	(37, 59, 63, 68)

*nd, not detected*.

Like spermidine, putrescine is found in virtually all foods of plant origin, and is particularly abundant in fruits and vegetables, notably citrus fruits (1,554 nmol/g) and green peppers (794 nmol/g) (9, 61). There are also high amounts of putrescine in wheat germ (705 nmol/g) and soybean sprouts (507 nmol/g) (37, 70).

The variability in polyamine contents in plant-derived products can be due to different factors, including their origin, growing conditions, harvesting, or storage. In this sense, different stress situations of the plant could affect the polyamine content. For example, polyamine levels in plants can increase in response to stress brought by high or low cultivation temperatures or drought (71). Studies show that the application of polyamines pre- and post-cultivation can compensate for the negative effects of cold or drought, thereby favoring germination, plant growth or survival (72–74). Another factor that could explain the high levels of putrescine in some vegetables, such as spinach and peas, is the presence of spoilage bacteria, mainly *Enterobacteriaceae* and *Clostridium spp*., which can form putrescine from its amino acid precursor ornithine by their amino acid-decarboxylase activity (12, 62, 75).

#### Food of Animal Origin

In animal-derived foods, like those of plant origin, the contents of polyamines are extremely variable (Table 3). Meat and its derivatives may contain high levels of spermidine and spermine, particularly the latter. Spermine values >148 nmol/g have been described in samples of beef, pork, chicken, cured ham, and sausages, without significant differences between fresh meats and derivatives (37, 63, 76, 77). In fish and its derivatives, the contents of spermine and spermidine are generally lower than in meat products, but clearly higher than in milk and eggs, where their levels are low. In most cheeses the values of spermine and spermidine are <10 and 69 nmol/g, respectively, with the exception of a blue cheese with a very high spermidine content (262 nmol/g) (37).

**Table 3:** Ranges of average polyamine content (nmol/g) in foods of animal origin.

**Food categories**	**Putrescine**	**Spermidine**	**Spermine**	**References**
**Fresh meat**				
Beef, veal, lamb, pork, chicken, rabbit, turkey, duck.	1.1–47	1–92	1–342	(9, 37, 53, 63, 68, 76–78)
**Cooked meat derivatives**				
Cooked ham, mortadella, wiener sausage, frankfurter, botifarra	4.5–11	15–28	11–99	(9, 68, 77)
**Cured and fermented meat derivatives**				
Dry-cured ham, dry-fermented sausage	5–1771	8–62	11–177	(63, 68, 76, 77, 79)
**Fresh fish and seafood products**				
White fish, cod, hake, salmon, tuna, sardine, shrimp, crab, calamari, oysters, scallops	nd−487	nd−167	nd−111	(37, 63, 68, 80)
**Semi-preserved and canned fish**				
Canned tuna, anchovies	1.1–47	6.2–28	12–53	(80, 81)
**Egg**	3.1–10	1–4	nd−1	(37, 63)
**Milk and dairy products**				
Milk, yogurt	nd−3	0.41–5	nd−4	(9, 68, 82)
**Cheese**				
Matured cheese, hard-ripened cheese, goat cheese, roquefort, gorgonzola, blue cheese, camembert, brie, comté, Swiss emmental, yellow cheese.	1.5–1470	nd−262	nd−17	(9, 37, 68, 82, 83)

*nd, not detected*.

In fresh products of animal origin (meat, fish, milk, and eggs) the putrescine contents are generally lower than in plant-derived foods. However, the highest levels of putrescine are found in products subjected to a fermentation process involving potentially aminogenic microorganisms. The wide range of putrescine contents could also be explained by the decarboxylase activity of spoilage bacteria. Studies show that the hygienic state of raw materials has an important influence on the formation of putrescine and other amines during the elaboration of different food products. For example, a greater accumulation of amines was reported in dry-fermented sausages when these were produced from raw materials of low microbial quality (78). This factor could also be responsible for increasing putrescine levels in long-maturing cheeses for whose manufacture the use of raw milk is an authorized practice. In this sense, the previous thermal treatment of milk is a useful tool, not only to guarantee the absence of pathogenic microorganisms but also to avoid the formation of putrescine and other biogenic amines, as it decreases a) the load of spoilage microorganisms with amino acid-decarboxylase capacity; b) the presence of free amino acid precursors by delaying proteolysis during ripening; and c) levels of the thermolabile pyridoxal phosphate, a necessary cofactor of the amino acid-decarboxylase enzyme (83).

#### Effects of Culinary Treatment

Culinary treatment can potentially decrease the polyamine content in foods by two possible mechanisms: (a) transfer to the cooking water or (b) due to the high temperatures reached in some types of cooking. The few studies evaluating the effect of culinary treatment on polyamines report variable results, depending on the type of cooking and the food studied. Polyamine contents after the boiling of certain vegetables (spinach, cauliflower, and potatoes) were significantly reduced by transfer to the cooking water, especially putrescine, as this is the most water-soluble polyamine. However, the same cooking process did not induce losses in other types of food (peppers, peas, and asparagus) (84). Another study found no significant differences in polyamine levels between raw and boiled vegetables (carrots, broccoli, cauliflower, and potatoes), although the low number of samples analyzed (two per food type) was a limiting factor (9). In meat subjected to a cooking process involving a large amount of water (stewing and boiling), no significant losses of spermidine and spermine were observed either (23, 53). In the case of some cooking techniques that involved higher temperatures (53) described that roasting, grilling, or frying produced losses of up to 60% of spermidine and spermine in chicken meat.

#### Antioxidant Potential of Polyamines in Food

Studies of the antioxidant role of polyamines in food are scarce compared to those in biological substrates. The protective effect of polyamines against oxidation when added to a lipid matrix has been demonstrated *in vitro*, mainly acting as metal chelators. A concentration-dependent antioxidant capacity was reported for spermine and spermidine (13). Later, Toro Funes et al. (16) also described an antioxidant effect for each of these polyamines at a wide range of concentrations (from 30 to 1,250 μg/mL). Specifically, spermine and spermidine delay the formation of peroxides and secondary oxidation compounds, the effects of spermine being greater due to a higher number of amino groups. In addition, these two studies showed that antioxidant activity of both polyamines is equal to or even higher than that of some antioxidant additives commonly used in foods, such as octyl gallate, alpha-tocopherol, ascorbyl palmitate, or tert-butylhydroquinone, among others.

Foods with high contents of polyamines, such as wheat germ, soya, mushroom, or citrus fruits, could be used as natural antioxidant ingredients in the form of powdered concentrates or polyamine-rich extracts. Prior to the use of these extracts or concentrates of polyamine-rich foods as natural antioxidants, effective and safe doses would need to be determined.

#### Analysis of Polyamines in Food

The analytical methodologies to determine polyamines in food are mainly based on the chromatographic separation coupled with distinct detection techniques due to their high resolution, sensitivity and versatility. Gas chromatography, thin-layer chromatography and high-performance liquid chromatography have been applied for the analysis of polyamines in food (85–87). Concretely, high or ultra high-performance liquid chromatography with ion-exchange columns or reverse-phase columns to separate polyamines are the most frequently reported techniques in the literature (88).

Different detection techniques coupled to chromatographic separation systems have been described such as UV, fluorescence and mass spectrometry. Polyamines have low absorption coefficients or quantum yields and require derivatization when the method involve UV or fluorescent detection. Chemical derivatization of these compounds can be carried out with a variety of reagents, mostly 5-dimethylamino-1-naphtalene-sulfonyl chloride (dansyl chloride) that forms stable compounds after reaction with both primary and secondary amino groups and *o*-phthaldialdehyde (OPA), which reacts rapidly (i.e., 30 seg) with primary amines. Amine derivatives can be formed before (pre-column), during (on-column) or after (post-column) the chromatographic separation. Pre-derivatization comprises a series of time- consuming manual steps and may introduce imprecision to the overall analytical procedure. Post-column derivatization has the advantage that it is automatically performed online, thereby avoiding sample manipulation and shortening the time required for the analysis (89). In recent years, the determination of polyamines through liquid chromatography coupled to mass spectrometry (MS) or tandem mass spectrometry (MS/MS) has emerged as an alternative analytical technique, very specific and sensitive and without the need of derivatization (52, 87, 88, 90).

Electrochemical sensors or biosensors are an alternative to the analytical procedures described above, being less expensive, less time-consuming, and analytically simpler, especially for routine screenings. Electrochemical biosensors usually consist on immobilized amino-oxidases, which catalyze the oxidative deamination of polyamines present in foods, and a working electrode that detects the production or the consumption of the redox species produced by the enzymatic activity. Different electrochemical sensors developed for the rapid determination of polyamines in food showed low detection limits and good selectivity toward these compounds (86).

### Polyamine Intake

The daily intake of polyamines has been estimated for different European countries, Japan and the United States (Table 4). The mean polyamine intake in the European adult population was estimated as 354 μmol/day, with differences among the member states, being lowest in the United Kingdom and highest in the countries of the Mediterranean area, Italy and Spain (91). Subsequent studies carried out in Mediterranean countries, such as Spain and Turkey, have estimated much lower intake values for these populations (94, 95), which could be partly related to a decrease in the consumption of plant-derived foods due to the progressive abandonment of the traditional Mediterranean diet observed in the last 20 years (96). The polyamine intake estimates for the adult population of Japan and the United States lie between the European mean and the values corresponding to the Mediterranean area. The only study estimating the intake in an adolescent population was carried out in Sweden (93) and the results were very similar to those previously reported for the Swedish adult population (91).

**Table 4:** Estimated average intake of polyamines (μmol/day) in different studies.

**References**	**Study population**	**Total polyamines**	**Putrescine**	**Spermidine**	**Spermine**
European Union^a^ (91)	Adults	353.6	211.9	87	54.7
United Kingdom		315.1	160.3	96.7	58.1
Finland		343.6	222.6	71.9	49.1
Sweden		362.9	250.5	70.0	42.3
Spain		384.3	211.7	103.1	69.5
Italy		387.7	247.4	83.6	56.7
Japan (63)	Children and adults J-NNS^b^	200	90	74	36
United States of America (92)	Adults 40 to 80 years	249.5	159.1	54.7	35.7
Sweden (93)	Adolescents 17 years	316	215.5	66	34.5
Turkey (94)	Adults 40 ± 19 years	139.9	93.1	33.1	13.7
Spain (95)	Adults ENALIA II^c^	170	–	–	–

a European Union: United Kingdom, Italy, Spain, Finland, Sweden, and the Netherlands.

b J-NNS: Nationwide nutrition survey in Japan.

c *Spanish national dietary survey in adults, elderly and pregnant women*.

The differences between intake estimates can be attributed not only to the different dietary patterns of each population, but also to the age group studied, the methodology of data collection and/or to the variability in food polyamine content. For example, the food consumption data used to estimate polyamine intake was obtained from published national surveys (Japan and Spain), a frequency-of-consumption questionnaire (United States), a 7 day food record (Sweden) and a 24 h dietary recall (Turkey). In some studies, the data on polyamine content were obtained from analyses carried out specifically for the intake estimation studies (63, 64, 95), whereas others used data already published in the literature (93, 94).

All the studies agree that the polyamine contributing most to the total intake is putrescine, mainly from the consumption of fruits and vegetables, or in Japan also from cereals and soy sauce. Fruits, vegetables and cereals are also the main sources of spermidine. The main origin of dietary spermine is meat and fish, except in Sweden, where it is vegetables and cereals.

At present there are no official recommendations for the daily intake of polyamines, but some suggestions have been made. Atiya Ali et al. (93) proposed an intake around of 540 μmol/day, taking into account the guidelines of a healthy diet that promotes a high consumption of fruits, vegetables and cereals (93). This estimate is two to three times higher than the intakes reported in the studies reviewed.

## Conclusions

There is extensive knowledge about the physiological functions of polyamines and their importance for human health. Several studies indicate the importance of dietary polyamines at different stages and situations of life, such as in the postnatal period or aging, when requirements are higher. In addition, the antioxidant and anti-inflammatory effects described for polyamines can play an important role in the prevention of chronic conditions such as cardiovascular diseases and diabetes. On the other hand, cancer is associated with high levels of polyamines, brought about by an alteration in their homeostasis.

The contents of polyamines in food, even within the same type, are highly variable. Breast milk provides the first dietary exposure to these compounds. Despite the scarcity and variability of available data, the content and profile of polyamines in breast milk are clearly different from those observed in infant formula. Among plant-derived foods, cereals, legumes and soybean derivatives are the categories with the highest contents of spermidine and spermine, whereas the highest putrescine levels are found in vegetables and fruits, especially citrus fruits. In animal-derived foods, meat and derivatives have the highest polyamine contents, with the exception of some cheeses. A range of factors could be responsible for the high variability in the polyamine content in food, notably origin and conditions of cultivation of plants, as well as the conditions of processing and storage. The wide range of putrescine contents could be also explained by the decarboxylase activity of spoilage or fermentative bacteria.

Polyamines have been associated with a high antioxidant activity in foods matrices, especially spermine. Therefore, foods rich in polyamines such as wheat germ, soybean, mushroom, or citrus fruits, in the form of extracts or concentrated powders, could be used as natural antioxidant ingredients. Such application will require previous studies to determine safety and effective dosage.

The few studies estimating polyamine intake have published highly variable results. This inconsistency could be attributed not only to the different diets of the studied populations, but also to methodological differences that could be related to the absence of consensus guidelines for the estimation of polyamine consumption. There are currently no official recommendations for daily polyamines intake, although some authors have proposed levels well above the intake estimates made in different countries. The dietary polyamine requirements in the different age groups should also be establish in order to be able to define a rich or low diet in polyamines.

## Author Contributions

All authors listed have made a substantial, direct and intellectual contribution to the work, and approved it for publication.

### Conflict of Interest Statement

The authors declare that the research was conducted in the absence of any commercial or financial relationships that could be construed as a potential conflict of interest.
